# Supplementary material for: The relationships between obesity and epilepsy: A systematic review with meta-analysis
Source: PLoS One. 2024 Aug 9;19(8):e0306175. doi: 10.1371/journal.pone.0306175 (PMC11315312; doi:10.1371/journal.pone.0306175)
Supplement: S1 File — (PDF) [file pone.0306175.s001.pdf]

Systematic review

Please select one of the options below to edit your record. Either option will create a new version of the record - the existing version will remain unchanged.

A list of fields that can be edited in an update can be found here

1. * Review title.

Give the title of the review in English

The relationships between obesity and Epilepsy:a meta-analysis

2. Original language title.

For reviews in languages other than English, give the title in the original language. This will be displayed with the English language title.

3. * Anticipated or actual start date.

Give the date the systematic review started or is expected to start.

25/06/2023

4. * Anticipated completion date.

Give the date by which the review is expected to be completed.

25/12/2023

5. * Stage of review at time of this submission.

This field uses answers to initial screening questions. It cannot be edited until after registration.

Tick the boxes to show which review tasks have been started and which have been completed.

Update this field each time any amendments are made to a published record.

The review has not yet started: No

Review stage	Started	Completed
Preliminary searches	Yes	No
Piloting of the study selection process	Yes	No
Formal screening of search results against eligibility criteria	No	No
Data extraction	No	No
Risk of bias (quality) assessment	No	No
Data analysis	No	No

Provide any other relevant information about the stage of the review here.

6. * Named contact.

The named contact is the guarantor for the accuracy of the information in the register record. This may be any member of the review team.

yuxuan li

Email salutation (e.g. "Dr Smith" or "Joanne") for correspondence:
Ms li

7. * Named contact email.

Give the electronic email address of the named contact.

liyuxuandldx@163.com

8. Named contact address

PLEASE NOTE this information will be published in the PROSPERO record so please do not enter private information, i.e. personal home address

Give the full institutional/organisational postal address for the named contact.

The First Affiliated Hospital of Dali University,Wanhua Road,Xiaguan Town,Dali city,Dali Bai Autonomous Prefecture,Yunnan Province，China

9. Named contact phone number.

Give the telephone number for the named contact, including international dialling code.

18408832102

10. * Organisational affiliation of the review.

Full title of the organisational affiliations for this review and website address if available. This field may be completed as 'None' if the review is not affiliated to any organisation.

The First Affiliated Hospital of Dali University

Organisation web address:

11. * Review team members and their organisational affiliations.

Give the personal details and the organisational affiliations of each member of the review team. Affiliation refers to groups or organisations to which review team members belong.
NOTE: email and country now MUST be entered for each person, unless you are amending a published record.

Ms yuxuan li. The First Affiliated Hospital of Dali University
Dr yun li. The First Affiliated Hospital of Dali University
Mr wang guo. The First Affiliated Hospital of Dali University

12. * Funding sources/sponsors.

Details of the individuals, organizations, groups, companies or other legal entities who have funded or sponsored the review.

no

Grant number(s)

State the funder, grant or award number and the date of award

13. * Conflicts of interest.

List actual or perceived conflicts of interest (financial or academic).

None

14. Collaborators.

Give the name and affiliation of any individuals or organisations who are working on the review but who are not listed as review team members. **NOTE: email and country must be completed for each person, unless you are amending a published record.**

15. * Review question.

State the review question(s) clearly and precisely. It may be appropriate to break very broad questions down into a series of related more specific questions. Questions may be framed or refined using PI(E)COS or similar where relevant.

At present, the correlation between obesity and epilepsy is still controversial, and further studies are needed to explore the correlation between the two. Specify prevention and treatment based on study results.

16. * Searches.

State the sources that will be searched (e.g. Medline). Give the search dates, and any restrictions (e.g. language or publication date). Do NOT enter the full search strategy (it may be provided as a link or attachment below.)

We will search for articles in four electronic databases including PubMed, EMBASE, Web of Science, and Cochrane Library. All the English publications until 25 June 2023 will be searched without any restriction of countries or article type. A reference list of all selected articles will independently be screened to identify additional studies left out in the initial search.

17. URL to search strategy.

Upload a file with your search strategy, or an example of a search strategy for a specific database, (including the keywords) in pdf or word format. In doing so you are consenting to the file being made publicly accessible.

Or provide a URL or link to the strategy. Do NOT provide links to your search **results**.

Do not make this file publicly available until the review is complete

18. * Condition or domain being studied.

Give a short description of the disease, condition or healthcare domain being studied in your systematic review.

Epilepsy is one of the most prevalent neurological diseases in clinical practice, with more than 70 million people affected around the world. This diseases is of great importance for the economic health of many countries. An estimated 25-30% of patients suffer from uncontrolled seizures, although on medication. Comorbidities of epilepsy are perceived as a conundrum in the treatment process.Epilepsy and obesity have attracted more and more attention, but the relationship between them is still controversial.further studies are needed to explore the correlation between the two. Specify prevention and treatment based on study results.

19. * Participants/population.

Specify the participants or populations being studied in the review. The preferred format includes details of both inclusion and exclusion criteria.

1) Age ≥18 years old; 2) Diagnosis of epilepsy according to the 2017 guidelines of the International League against Epilepsy (ILAE).

20. * Intervention(s), exposure(s).

Give full and clear descriptions or definitions of the interventions or the exposures to be reviewed. The preferred format includes details of both inclusion and exclusion criteria.

no

21. * Comparator(s)/control.

Where relevant, give details of the alternatives against which the intervention/exposure will be compared (e.g. another intervention or a non-exposed control group). The preferred format includes details of both inclusion and exclusion criteria.

healthy person

22. * Types of study to be included.

Give details of the study designs (e.g. RCT) that are eligible for inclusion in the review. The preferred format includes both inclusion and exclusion criteria. If there are no restrictions on the types of study, this should be stated.

Case-Control Studies, cohort studies, Cross-Sectional Studies, etc.

23. Context.

Give summary details of the setting or other relevant characteristics, which help define the inclusion or exclusion criteria.

24. * Main outcome(s).

Give the pre-specified main (most important) outcomes of the review, including details of how the outcome is defined and measured and when these measurement are made, if these are part of the review inclusion criteria.

Body Mass Index

Measures of effect
relative risks(RR), odds ratios(OR), risk difference(RD), etc.

25. * Additional outcome(s).

List the pre-specified additional outcomes of the review, with a similar level of detail to that required for main outcomes. Where there are no additional outcomes please state 'None' or 'Not applicable' as appropriate to the review

age, sex, years of illness, seizure type, body mass index, and study type, etc.

Measures of effect
relative risks(RR), odds ratios(OR), risk difference(RD), etc.

26. * Data extraction (selection and coding).

Describe how studies will be selected for inclusion. State what data will be extracted or obtained. State how this will be done and recorded.

Two authors independently extracted the data from the included studies. Any uncertainty was resolved by discussion between the authors. Data extracted from eligible literature included:the first author, year of publication, study design, country, population, age, gender, obesity presence, Body Mass Index, Seizure type, medicine, type of statistical analysis, odds ratio (OR), 95% confidence interval (95% CI), P-value and author conclusions, Univariate analysis, and multivariate analysis results.

27. * Risk of bias (quality) assessment.

State which characteristics of the studies will be assessed and/or any formal risk of bias/quality assessment tools that will be used.

The quality of all eligible studies was assessed using the Newcastle-Ottawa Scale (NOS) tool for cohort and case-control studies. The NOS tool evaluates a study based on the following aspects: the selection of study groups, the comparability of study groups and the assessment of exposure and outcome.

28. * Strategy for data synthesis.

Describe the methods you plan to use to synthesise data. This **must not be generic text** but should be **specific to your review** and describe how the proposed approach will be applied to your data.
If meta-analysis is planned, describe the models to be used, methods to explore statistical heterogeneity, and software package to be used.

The analysis was performed using the Cochrane Collaboration' s Review Manager Soft-ware (RevMan, version 5.3). We used the mean difference as the effect indicator, providing each effect-size point estimate and 95% confidence interval(CI). The Q test and I^2 statistics were used to perform the heterogeneity tests. For p 0.05 and $I^2<50$ %, we considered that there was no significant heterogene-ity and selected a fixed-effect model. For p<0.05 and 50%, subgroup analysis was used to determine the cause of heterogeneity. If heterogeneity could not be eliminated, we used a random-effects model.

29. * Analysis of subgroups or subsets.

State any planned investigation of 'subgroups'. Be clear and specific about which type of study or participant will be included in each group or covariate investigated. State the planned analytic approach.

Subgroup analysis was performed for general age, sex, years of illness, seizure type, medicine, body mass index, and study type

30. * Type and method of review.

Select the type of review, review method and health area from the lists below.

Type of review

Cost effectiveness	No
Diagnostic	No
Epidemiologic	No
Individual patient data (IPD) meta-analysis	No
Intervention	No
Living systematic review	No
Meta-analysis	Yes
Methodology	No
Narrative synthesis	No
Network meta-analysis	No
Pre-clinical	No
Prevention	No
Prognostic	No
Prospective meta-analysis (PMA)	No
Review of reviews	No
Service delivery	No

Synthesis of qualitative studies	No
Systematic review	Yes
Other	No

Health area of the review

Alcohol/substance misuse/abuse	No
Blood and immune system	No
Cancer	No
Cardiovascular	No
Care of the elderly	No
Child health	No
Complementary therapies	No
COVID-19	No
Crime and justice	No
Dental	No
Digestive system	No
Ear, nose and throat	No
Education	No
Endocrine and metabolic disorders	No
Eye disorders	No
General interest	No
Genetics	No
Health inequalities/health equity	No
Infections and infestations	No
International development	No
Mental health and behavioural conditions	No
Musculoskeletal	No
Neurological	Yes
Nursing	No
Obstetrics and gynaecology	No

Oral health	No
Palliative care	No
Perioperative care	No
Physiotherapy	No
Pregnancy and childbirth	No
Public health (including social determinants of health)	No
Rehabilitation	No
Respiratory disorders	No
Service delivery	No
Skin disorders	No
Social care	No
Surgery	No
Tropical Medicine	No
Urological	No
Wounds, injuries and accidents	No
Violence and abuse	No

31. Language.

Select each language individually to add it to the list below, use the bin icon to remove any added in error.

English

There is not an English language summary

32. * Country.

Select the country in which the review is being carried out. For multi-national collaborations select all the countries involved.

China

33. Other registration details.

Name any other organisation where the systematic review title or protocol is registered (e.g. Campbell, or The Joanna Briggs Institute) together with any unique identification number assigned by them.
If extracted data will be stored and made available through a repository such as the Systematic Review Data Repository (SRDR), details and a link should be included here. If none, leave blank.

34. Reference and/or URL for published protocol.

If the protocol for this review is published provide details (authors, title and journal details, preferably in Vancouver format)

No I do not make this file publicly available until the review is complete

35. Dissemination plans.

Do you intend to publish the review on completion?

Yes

36. Keywords.

Give words or phrases that best describe the review. Separate keywords with a semicolon or new line. Keywords help PROSPERO users find your review (keywords do not appear in the public record but are included in searches). Be as specific and precise as possible. Avoid acronyms and abbreviations unless these are in wide use.

37. Details of any existing review of the same topic by the same authors.

If you are registering an update of an existing review give details of the earlier versions and include a full bibliographic reference, if available.

38. * Current review status.

Update review status when the review is completed and when it is published.
New registrations must be ongoing so this field is not editable for initial submission.

Review_Ongoing

39. Any additional information.

Provide any other information relevant to the registration of this review.

40. Details of final report/publication(s) or preprints if available.

Leave empty until publication details are available OR you have a link to a preprint (NOTE: this field is not editable for initial submission).
List authors, title and journal details preferably in Vancouver format.
