# Supplementary material for: The relationships between obesity and epilepsy: A systematic review with meta-analysis
Source: PLoS One. 2024 Aug 9;19(8):e0306175. doi: 10.1371/journal.pone.0306175 (PMC11315312; doi:10.1371/journal.pone.0306175)
Supplement: S2 File — (DOCX) [file pone.0306175.s002.docx]

| **Section and Topic** | **Item #** | **Checklist item** | **Location where item is reported** |
| --- | --- | --- | --- |
| **TITLE** | | |  |
| Title | 1 | The report is identified as a systematic review and meta-analysis. |  |
| **ABSTRACT** | | |  |
| Abstract | 2 | The structured abstract includes objective, research design,and methods, results and conclusion. |  |
| **INTRODUCTION** | | |  |
| Rationale | 3 | Meta-analysis is a statistical method used to compare and synthesize the results of studies on the same scientific question. The significance of the conclusions depends on the quality of the included studies. It is often used for quantitative pooled analysis in systematic reviews. By integrating all relevant studies, health care outcomes can be estimated more accurately than in a single study, and it is useful to explore the consistency of evidence across studies and differences between studies. When the results of multiple studies are inconsistent or have no statistical significance, the statistical analysis results close to the real situation can be obtained by using Meta analysis. |  |
| Objectives | 4 | There is still controversy about whether obesity is a comorbidities of epilepsy. Therefore, this purpose of this study was to investigate the relationship between epilepsy and obesity. |  |
| **METHODS** | | |  |
| Eligibility criteria | 5 | The predefined inclusion criteria were as follows:1)Inclusion of case-control,cohort,randomized controlled trial,and cross-sectional studies;2)Segregation of subjects into epileptic patients and healthy controls;3)Obesity as the outcome measure;4)Availability of comprehensive data;5)Publication in English.The exclusion criteria were as follows:1)Exclusion of animal experiments,reviews,and other types of studies;2)Absence of a healthy control group;3)Incomplete data;4)Unextractable or unconvertible data;5)Low quality,indicated by an Agency for Healthcare Research and Quality(AHRQ) score of 5 or lower,or a Newcastle-Ottawa Scale (NOS) score less than 3.The subjects included in the study included adults and children,and the diagnostic criteria for obesity were used at different ages.Criteria for adult obesity:1)underweight,body mass index (BMI)<18.5 kg/m^2^;2)Normal, BMI 18.5-24.9kg/m^2^;3)Overweight,BMI 25-29.9kg/m^2^;4)Obesity,BMI ≥30kg/m^2^.Criteria for childhood obesity,dividing BMI percentiles into the following categories:1)Obesity:above the 95th percentile for BMI age;2)Overweight: 85th percentile of BMI age to 95th percentile of BMI age;3)Healthy weight:10th percentile of BMI age to 85th percentile of BMI age;4)Underweight: below the 10th percentile for BMI age.In this study,outcome measure obesity was defined as BMI≥25kg/m^2^ in adults and above the 85th percentile of BMI age in children. |  |
| Information sources | 6 | This study searched electronic databases from the Cochrane Library, PubMed, Web of Sciences and Embase until February 10,2024. |  |
| Search strategy | 7 | Details can be found in S4 File 4.Search Strategy. |  |
| Selection process | 8 | Two reviewers independently assessed study eligibility.They used endnot for literature management. |  |
| Data collection process | 9 | Two reviewers independently extracted data.They used endnot for literature management and excel spreadsheets for data extraction. |  |
| Data items | 10a | We used obesity as an outcome index to extract the number of obese and non-obese people in epilepsy group and healthy control group. |  |
|  | 10b | In this study, outcome measure obesity was defined as BMI≥25kg /m2 for adults and BMI age above the 85th percentile for children. Eliminate any articles with missing or unclear information. |  |
| Study risk of bias assessment | 11 | Methodological quality assessment of case-control studies followed the Newcastle-Ottawa Scale (NOS),with NOS scores categorized as 0-3 (low quality),4-6 (medium quality),and 7-9 (high quality).The cross-sectional study used criteria recommended by the Agency for Health Care Research and Quality (AHRQ) for quality assessment,with AHRQ scores categorized as 0-5 (low quality),6-7 (medium quality),and 8-11 (high quality).Two reviewers independently assessed the risk of bias. |  |
| Effect measures | 12 | In this study,outcome measure obesity was defined as BMI≥25kg/m^2^ for adults and BMI age above the 85th percentile for children. |  |
| Synthesis methods | 13a | Two reviewers independently screened the articles and extracted data according to inclusion and exclusion criteria. In case of disputes, the third reviewer will make the assessment. |  |
|  | 13b | Use excel tables for data extraction. Try to ask the author for the missing summary statistics. The data were transformed according to the diagnostic criteria of obesity. |  |
|  | 13c | The data were transformed and combined according to the diagnostic criteria for obesity. |  |
|  | 13d | We used RevMan 5.3 for our meta-analysis.We employed the mean difference as the effect measure,presenting each effect size with a corresponding 95% confidence interval (CI).To assess heterogeneity,we employed the Q test and calculated the I^2^ statistic.In cases where p>0.05 and I^2^<50%,we regarded heterogeneity as nonsignificant and opted for the fixed-effect model.When p<0.05 and I^2^>50%,we conducted subgroup analysis to identify the source of heterogeneity.If heterogeneity persisted,we employed a random-effects model. |  |
|  | 13e | We used subgroup analysis to explore possible reasons for the heterogeneity among the findings. |  |
|  | 13f | Sensitivity analysis was performed to observe and look for possible heterogeneity in this study,involving the substitution of the fixed-effects model with a random-effects model(S4 Fig 4),revealing no significant heterogeneity(p=0.01,I^2^=49%).Additionally,we conducted sensitivity analysis by excluding studies with the highest weight(S5 Fig 5),which did reveal heterogeneity(p<0.05,I^2^=52%),suggesting instability in the meta-analysis results. |  |
| Reporting bias assessment | 14 | To assess publication bias,we employed funnel plots(S3 Fig 3 in the attachment). |  |
| Certainty assessment | 15 | The 95% confidence interval of meta-analysis was used to evaluate the certainty of the evidence body. |  |
| RESULTS | | |  |
| Study selection | 16a | Initially,1497 articles were retrieved from four electronic databases using our search strategy,resulting in 313 articles after removing duplicates.Upon title and abstract evaluation,837 irrelevant articles were excluded,leaving 214 articles for further examination.After full-text review,100 studies lacked healthy controls,4 articles were inaccessible in full,49 studies had unextractable or untranslatable data,and 43 were unrelated studies,all of which were excluded.The review process is depicted in S1 Fig 1. |  |
|  | 16b | After full-text review,100 studies lacked healthy controls,4 articles were inaccessible in full,49 studies had unextractable or untranslatable data,and 43 were unrelated studies,all of which were excluded.The review process is depicted in S1 Fig 1. |  |
| Study characteristics | 17 | The characteristics of each included study are shown in S1 Table 1 of the manuscript. |  |
| Risk of bias in studies | 18 | The Newcastle-Ottawa Scale (NOS) assessed the quality of included case-control studies，with NOS scores categorized as 0-3 (low quality), 4-6 (medium quality), and 7-9 (high quality).The cross-sectional study used criteria recommended by the Agency for Health Care Research and Quality (AHRQ) for quality assessment，with AHRQ scores categorized as 0-5(low quality), 6-7 (medium quality), and 8-11 (high quality).Specific information on the quality assessment of the included studies can be found in S1 Table 1 of the manuscript and S5 File 5 of the Supporting Information. |  |
| Results of individual studies | 19 | All results for each study are shown in Figure 2,4,5,6,7,8 in the annex. |  |
| Results of syntheses | 20a | In total,17 studies were included,comprising 13 of high quality and 4 of medium quality.Specific information on the quality assessment of the included studies can be found in S1 Table 1 of the manuscript and S5 File 5 of the Supporting Information.For each synthesis, the characteristics of the contributed research are shown in S1 Table 1 of the manuscript and S2 Fig 2 in the annex. |  |
|  | 20b | In summary, this study detected heterogeneity through sensitivity analysis by excluding studies with the highest weight, highlighting the need for caution in interpreting the results due to potential instability. To explore the sources of heterogeneity, subgroup analysis was conducted, revealing age, publication years of included articles, and the level of economic development as potential factors. The study encompassed both adults and children, acknowledging variations in the prevalence of post-epileptic obesity across different age groups, which could contribute to heterogeneity among studies. Nonetheless, subgroup analysis still indicates a correlation between epilepsy and obesity in both adults and children. Additionally, considering that the majority of included studies were published a decade ago and primarily conducted in developed countries, there is a possibility of time bias in the results, while economic development could also influence obesity outcomes. Subgroup analysis further highlighted that publication years of articles and economic development at research sites contributed to the observed heterogeneity. |  |
|  | 20c | 1.Subgroup analysis was based on the age of the subjects(S6 Fig6)  ①Studies involving adults did not find significant heterogeneity(p=0.05, I2=45%) and showed statistically significant differences in obesity(OR=1.24,95%CI:1.15-1.34,p<0.01).②Conversely,there was significant heterogeneity in the results of the study involving children(p=0.06,I2=53%),with the results demonstrating a statistically significant difference in obesity(OR=1.47,95%CI:1.25-1.72,p<0.01).The subgroup difference test(p=0.07,I2=69.1%) indicated that Age was associated with the heterogeneity observed in this meta-analysis.  2.Subgroup analysis based on the year of publication (S7 Fig 7)  ① There was no significant heterogeneity in the results of articles published within the last 10 years (p=0.08,I2=49%),and the results showed a significant difference in obesity between epilepsy patients and healthy subjects (OR=1.48,95%CI:1.27-1.72,p<0.01).Results published more than 10 years ago did not find significant heterogeneity (p=0.06,I2=44%),but showed a statistically significant difference in obesity (OR=1.23,95%CI:1.14-1.34,p<0.01).Subgroup difference tests (p=0.04,I2=76.7%) showed that the year of publication was associated with the observed heterogeneity.  3. Subgroup analysis based on economic development level (S8 Fig 8)  ① In the developed countries,there was significant heterogeneity (p=0.02,I2=54%),and the difference in obesity was statistically significant (OR=1.26,95%CI:1.17-1.35,p<0.01).In contrast,studies from developing countries found no significant heterogeneity (p=0.28,I2=21%) and showed statistically significant differences in obesity (OR=1.81,95%CI:1.35-2.42,p<0.01).A subgroup difference test (p=0.02,I2=81.7%) showed that the level of published economic development was associated with the heterogeneity observed in this study. |  |
|  | 20d | Sensitivity analysis was performed to observe and look for possible heterogeneity in this study,involving the substitution of the fixed-effects model with a random-effects model(S4 Fig 4),revealing no significant heterogeneity(p=0.01,I2=49%).Additionally,we conducted sensitivity analysis by excluding studies with the highest weight(S5 Fig 5),which did reveal heterogeneity(p<0.05,I2=52%),suggesting instability in the meta-analysis results. |  |
| Reporting biases | 21 | There was no significant change in the comprehensive evaluation results after deleting each article one by one. |  |
| Certainty of evidence | 22 | The evidence for each outcome assessed was assessed using confidence. As shown in attached S2 Fig 2. |  |
| DISCUSSION | | |  |
| Discussion | 23a | There is a growing emphasis on enhancing the quality of life for individuals with epilepsy and understanding the comorbidities associated with this condition. Research has identified that epilepsy patients are susceptible to cognitive disorders, depression, obesity, and endocrine disturbances. However, the link between epilepsy and obesity remains a subject of debate, and the underlying mechanisms connecting these two conditions remain unclear. The findings from this meta-analysis indicate a higher prevalence of obesity among individuals with epilepsy compared to healthy individuals.Currently, the predominant focus of research lies in examining the impact of antiepileptic drugs on obesity, with particular attention to sodium valproate. Sodium valproate is commonly used to treat a range of seizures, including simple or complex absence seizures, myoclonic seizures, and severe seizures, either alone or in combination. Its cost-effectiveness has contributed to its widespread clinical usage. Numerous studies have established that sodium valproate is associated with several adverse effects, including elevated levels of leptin, insulin resistance, increased leptin/adiponectin ratio, hyperinsulinemia, elevated body mass index (BMI), increased blood lipid levels, reduced carnitine, significant reductions in bone mineral density, elevated liver enzyme levels, menstrual irregularities, altered reproductive hormone function, mitochondrial dysfunction, endocrine disturbances, and an increased risk of polycystic ovary syndrome among women of reproductive age [16][24-31]. Furthermore, some studies have suggested that sodium valproate-induced obesity and weight gain might be linked to CD36 and PPARγ polymorphisms, with these genetic factors potentially serving as predictive markers for sodium valproate-induced obesity in Chinese Han epilepsy patients [32]. However, research concerning the effects of other antiepileptic drugs on obesity remains limited and contentious. Existing evidence suggests that fenfluramine, zonisamide, and topiramate may lead to weight reduction, while carbamazepine and oxcarbamazepine tend to promote weight gain [33-37]. Horizontal genetic studies have found that SLC13A5 epilepsy is caused by newborn SLC13A5 mutations. The sodium-dependent citric acid transporter NaCT encoded by SLC13A5 may be a potential target for anti-obesity intervention. [38]. It is conceivable that future research may unveil more genetic connections between epilepsy and obesity, potentially opening new avenues for the treatment of epilepsy. In clinical practice, it is essential to monitor the impact of various antiepileptic drugs on blood lipid profiles and body weight while also considering the cardiovascular and cerebrovascular risks associated with these medications.  （This has been described in the discussion of the article.） |  |
|  | 23b | Currently, there is a scarcity of large-scale prospective studies. Additional clinical investigations are warranted to delve deeper into whether obesity is a comorbidity of epilepsy and whether obesity can potentially trigger epilepsy. |  |
|  | 23c | This study is subject to several limitations. Firstly, the predominance of case-control and cross-sectional studies, with limited inclusion of cohort studies and randomized controlled trials, impedes the comprehensive establishment of a causal relationship between epilepsy and obesity. Secondly, heterogeneity among studies may arise due to variations in cultural backgrounds, regions, and measurement tools for outcome indicators, potentially impacting research findings. Additionally, factors such as anti-epileptic drugs, dosage regimens, patient activity levels, disease duration, and seizure frequency mentioned in the literature also exert influence on obesity; however, insufficient data hindered meta-analysis. Large-scale prospective studies are warranted to further investigate whether obesity acts as a comorbidity of epilepsy or if it may serve as a trigger for epilepsy onset. Future endeavors should prioritize rational BMI management and individualized medication strategies for patients with epilepsy. These findings hold significant value in highlighting cardiovascular risks among patients while preventing the occurrence and progression of epilepsy while identifying novel therapeutic targets. |  |
|  | 23d | The results of this meta-analysis suggest that epilepsy patients are more likely to develop obesity than healthy people, and the problem of post-epilepsy obesity needs to be paid more attention in clinical practice. In future clinical practice, we suggest monitoring the effects of different antiepileptic drugs on blood lipid profile and body weight. Concern about the cardiovascular and cerebrovascular risks of antiepileptic drugs. In the future, more prospective large sample studies are needed to further explore the correlation between epilepsy and obesity and its influencing factors, whether obese people are more prone to epilepsy and the related pathogenesis of obesity and epilepsy. |  |
| **OTHER INFORMATION** | | |  |
| Registration and protocol | 24a | This study has been registered with Prospero(CRD42023439530).Registration information：register name：Yu-xuan Li,registration number：liyuxuandldx@163.com. |  |
|  | 24b | This study has been registered with Prospero(CRD42023439530).You can access the review agreement through Prospero or view a PDF attached to our supporting information (S1 File 1). |  |
|  | 24c | We will explain in detail the changes to the registration content.(1)The registration was titled "The relationships between obesity and Epilepsy:a meta-analysis", It is now changed to "The relationships between obesity and Epilepsy:A systematic review with meta-analysis", as not only a meta-analysis but also a systematic review is performed in this paper;(2)The team initially comprised 3 members but eventually expanded to 5 members due to the heavy workload of literature retrieval, screening, and extraction. |  |
| Support | 25 | The author(s) received no specific funding for this work. |  |
| Competing interests | 26 | The authors have declared that no competing interests exist. |  |
| Availability of data, code and other materials | 27 | Data collection form templates and all data used for analysis are publicly available. This information can be found in the article (S1Table 1 in the manuscript). |  |

*From:*  Page MJ, McKenzie JE, Bossuyt PM, Boutron I, Hoffmann TC, Mulrow CD, et al. The PRISMA 2020 statement: an updated guideline for reporting systematic reviews. BMJ 2021;372:n71. doi: 10.1136/bmj.n71

For more information, visit: <http://www.prisma-statement.org/>
