# Supplementary material for: The relationships between obesity and epilepsy: A systematic review with meta-analysis
Source: PLoS One. 2024 Aug 9;19(8):e0306175. doi: 10.1371/journal.pone.0306175 (PMC11315312; doi:10.1371/journal.pone.0306175)
Supplement: S4 File — (DOCX) [file pone.0306175.s004.docx]

**search strategy**

This study followed the PRISMA guidelines for systematic review and meta-analysis.It was registered with Prospero (CRD42023439530) and involved an extensive search of electronic databases,including the Cochrane Library,PubMed, Web of Science,and Embase,up to February 10,2024.The search incorporated keywords such as"Epilepsy","Obesity","Case-Control Studies","cohort studies","Rando-mized Controlled Trial" and "Cross-Sectional Studies".Develop comprehensive s-earch strategies with PubMed MeSH.

The specific search strategy of this study is as follows, taking pubmed as an example.

**1、Case-Control Studies**

Search: (((((((((((((("Epilepsy"[Mesh]) OR (Epilepsy[Title/Abstract])) OR (Epilepsies[Title/Abstract])) OR (Seizure Disorder[Title/Abstract])) OR (Seizure Disorders[Title/Abstract])) OR (Awakening Epilepsy[Title/Abstract])) OR (Epilepsy, Awakening[Title/Abstract])) OR (Epilepsy, Cryptogenic[Title/Abstract])) OR (Cryptogenic Epilepsies[Title/Abstract])) OR (Cryptogenic Epilepsy[Title/Abstract])) OR (Epilepsies, Cryptogenic[Title/Abstract])) OR (Aura[Title/Abstract])) OR (Auras[Title/Abstract])) AND ("Obesity"[Mesh])) AND ((((((((((((((((((((((((((((((((((((((((((Case-Control Studies) OR (Case-Control Study)) OR (Studies, Case-Control)) OR (Study, Case-Control)) OR (Case-Comparison Studies)) OR (Case Comparison Studies)) OR (Case-Comparison Study)) OR (Studies, Case-Comparison)) OR (Study, Case-Comparison)) OR (Case-Compeer Studies)) OR (Studies, Case-Compeer)) OR (Case-Referrent Studies)) OR (Case Referrent Studies)) OR (Case-Referrent Study)) OR (Studies, Case-Referrent)) OR (Study, Case-Referrent)) OR (Case-Referent Studies)) OR (Case Referent Studies)) OR (Case-Referent Study)) OR (Studies, Case-Referent)) OR (Study, Case-Referent)) OR (Case-Base Studies)) OR (Case Base Studies)) OR (Studies, Case-Base)) OR (Studies, Case-Base)) OR (Case Control Study)) OR (Studies, Case Control)) OR (Study, Case Control)) OR (Nested Case-Control Studies)) OR (Case-Control Studies, Nested)) OR (Case-Control Study, Nested)) OR (Nested Case Control Studies)) OR (Nested Case-Control Study)) OR (Studies, Nested Case-Control)) OR (Study, Nested Case-Control)) OR (Matched Case-Control Studies)) OR (Case-Control Studies, Matched)) OR (Case-Control Study, Matched)) OR (Matched Case Control Studies)) OR (Matched Case-Control Study)) OR (Studies, Matched Case-Control)) OR (Study, Matched Case-Control))

**2、****Cohort Studies**

Search: (((((((((((((("Epilepsy"[Mesh]) OR (Epilepsy[Title/Abstract])) OR (Epilepsies[Title/Abstract])) OR (Seizure Disorder[Title/Abstract])) OR (Seizure Disorders[Title/Abstract])) OR (Awakening Epilepsy[Title/Abstract])) OR (Epilepsy, Awakening[Title/Abstract])) OR (Epilepsy, Cryptogenic[Title/Abstract])) OR (Cryptogenic Epilepsies[Title/Abstract])) OR (Cryptogenic Epilepsy[Title/Abstract])) OR (Epilepsies, Cryptogenic[Title/Abstract])) OR (Aura[Title/Abstract])) OR (Auras[Title/Abstract])) AND ("Obesity"[Mesh])) AND ((((((((((((((((((((((((((((((((((Cohort Studies) OR (Cohort Study[Title/Abstract])) OR (Studies, Cohort[Title/Abstract])) OR (Study, Cohort[Title/Abstract])) OR (Concurrent Studies[Title/Abstract])) OR (Studies, Concurrent[Title/Abstract])) OR (Concurrent Study[Title/Abstract])) OR (Study, Concurrent[Title/Abstract])) OR (Closed Cohort Studies[Title/Abstract])) OR (Cohort Studies, Closed[Title/Abstract])) OR (Closed Cohort Study[Title/Abstract])) OR (Cohort Study, Closed[Title/Abstract])) OR (Study, Closed Cohort[Title/Abstract])) OR (Studies, Closed Cohort[Title/Abstract])) OR (Birth Cohort Studies[Title/Abstract])) OR (Birth Cohort Study[Title/Abstract])) OR (Cohort Studies, Birth[Title/Abstract])) OR (Cohort Study, Birth[Title/Abstract])) OR (Studies, Birth Cohort[Title/Abstract])) OR (Study, Birth Cohort[Title/Abstract])) OR (Analysis, Cohort[Title/Abstract])) OR (Analyses, Cohort[Title/Abstract])) OR (Cohort Analyses[Title/Abstract])) OR (Cohort Analysis[Title/Abstract])) OR (Cohort Analysis[Title/Abstract])) OR (Cohort Studies, Historical[Title/Abstract])) OR (Cohort Study, Historical[Title/Abstract])) OR (Historical Cohort Study[Title/Abstract])) OR (Study, Historical Cohort[Title/Abstract])) OR (Studies, Historical Cohort[Title/Abstract])) OR (Incidence Studies[Title/Abstract])) OR (Incidence Study[Title/Abstract])) OR (Studies, Incidence[Title/Abstract])) OR (Study, Incidence[Title/Abstract]))

**3、****Randomized Controlled Trial**

Search: (((((((((((((("Epilepsy"[Mesh]) OR (Epilepsy[Title/Abstract])) OR (Epilepsies[Title/Abstract])) OR (Seizure Disorder[Title/Abstract])) OR (Seizure Disorders[Title/Abstract])) OR (Awakening Epilepsy[Title/Abstract])) OR (Epilepsy, Awakening[Title/Abstract])) OR (Epilepsy, Cryptogenic[Title/Abstract])) OR (Cryptogenic Epilepsies[Title/Abstract])) OR (Cryptogenic Epilepsy[Title/Abstract])) OR (Epilepsies, Cryptogenic[Title/Abstract])) OR (Aura[Title/Abstract])) OR (Auras[Title/Abstract])) AND ("Obesity"[Mesh])) AND ("Randomized Controlled Trial" [Publication Type])

**4、Cross-Sectional Studies**

Search: (((((((((((((("Epilepsy"[Mesh]) OR (Epilepsy[Title/Abstract])) OR (Epilepsies[Title/Abstract])) OR (Seizure Disorder[Title/Abstract])) OR (Seizure Disorders[Title/Abstract])) OR (Awakening Epilepsy[Title/Abstract])) OR (Epilepsy, Awakening[Title/Abstract])) OR (Epilepsy, Cryptogenic[Title/Abstract])) OR (Cryptogenic Epilepsies[Title/Abstract])) OR (Cryptogenic Epilepsy[Title/Abstract])) OR (Epilepsies, Cryptogenic[Title/Abstract])) OR (Aura[Title/Abstract])) OR (Auras[Title/Abstract])) AND ("Obesity"[Mesh])) AND (((((((((((((((((((((((((Cross-Sectional Studies) OR (Cross-Sectional Study)) OR (Studies, Cross-Sectional)) OR (Study, Cross-Sectional)) OR (Cross Sectional Analysis)) OR (Analyses, Cross Sectional)) OR (Cross Sectional Analyses)) OR (Disease Frequency Surveys)) OR (Cross-Sectional Survey)) OR (Cross Sectional Survey)) OR (Cross-Sectional Surveys)) OR (Survey, Cross-Sectional)) OR (Surveys, Cross-Sectional)) OR (Surveys, Disease Frequency)) OR (Disease Frequency Survey)) OR (Survey, Disease Frequency)) OR (Analysis, Cross-Sectional)) OR (Analyses, Cross-Sectional)) OR (Analysis, Cross Sectional)) OR (Cross-Sectional Analyses)) OR (Cross-Sectional Analyses)) OR (Prevalence Studies)) OR (Prevalence Study)) OR (Studies, Prevalence)) OR (Study, Prevalence))
