# Supplementary material for: The relationships between obesity and epilepsy: A systematic review with meta-analysis
Source: PLoS One. 2024 Aug 9;19(8):e0306175. doi: 10.1371/journal.pone.0306175 (PMC11315312; doi:10.1371/journal.pone.0306175)
Supplement: S5 File — (DOCX) [file pone.0306175.s005.docx]

**Quality Evaluation**

The Newcastle-Ottawa Scale (NOS) assessed the quality of included case-control studies.The cross-sectional study used criteria recommended by the Agency for Health Care Research and Quality (AHRQ) for quality assessment.

**Table 1. Risk levels of bias in case-control studies.**

| **item** | | **Pylvänen,V,2002** | **Pylvänen,V,2003** | **El-Khayat,H.A,2004** | **Pylvänen,V,2006** | **Mania M,2011** | **Ayyagari,M,2012** | **Z.S.Daniels,BA,2015** | **Inaloo S,2020** |
| --- | --- | --- | --- | --- | --- | --- | --- | --- | --- |
| **SELECTION** | 1)Is the Case Definition Adequate? | * | * | * | * | * | * | * | * |
|  | 2) Representativeness of the Cases | * | * | * | * | * | * | * | * |
|  | 3) Selection of Controls | * | * | * | / | / | / | * | * |
|  | 4) Definition of Controls | * | * | * | * | * | * | * | * |
| **COMPARABILITY** | 1) Comparability of Cases and Controls on the Basis of the Design or Analysis | * | * | * | / | ** | * | ** | ** |
| **EXPOSURE** | 1) Ascertainment of Exposure | * | * | * | * | * | * | * | * |
|  | 2)Whether case and control exposures were determined using the same method | * | * | * | * | * | * | * | * |
|  | 3) Non-Response Rate | / | / | * | * | / | / | / | * |
|  | total points | 7 | 7 | 8 | 6 | 7 | 6 | 8 | 9 |
| Note:Each entry on "Selection" and "exposure" for each study can have up to one "*". Entries on "comparability" can have up to two "*".The total score is 9 points, and studies with scores of 0 to 3, 4 to 6, and 7 to 9 correspond to low, medium, and high quality studies respectively. | | | | | | | | | |

As shown in Table 1, among the 8 case-control studies included in this meta-analysis, 6 were of high quality and 2 were of medium quality.

**Table 2. Risk levels of bias in cross-sectional studies.**

| **item** | **Marquez AV,2003** | **Kobau R,2004** | **Wong J,2006** | **Elliott JO,2008** | **Hinnell,C,2010** | **Arya,R,2016** | **Khuda,I.E,2022** | **Tadegew Adane,2023** | **Buro AW,2023** | **George LJ,2022** |
| --- | --- | --- | --- | --- | --- | --- | --- | --- | --- | --- |
| 1) Define the source of information (survey, record review) | yes | yes | yes | yes | yes | yes | yes | yes | yes | yes |
| 2) List inclusion and exclusion criteria for exposed and unexposed subjects (cases and controls) or refer to previous publications | yes | yes | yes | yes | no | yes | yes | yes | yes | yes |
| 3) Indicate time period used for identifying patients | yes | yes | yes | yes | yes | yes | yes | yes | yes | yes |
| 4) Indicate whether or not subjects were consecutive if not population-based | yes | yes | yes | yes | yes | yes | yes | no | yes | yes |
| 5) Indicate if evaluators of subjective components of study were masked to other aspects of the status of the participants | no | no | no | no | no | no | no | no | no | no |
| 6) Describe any assessments undertaken for quality assurance purposes (e.g., test/retest of primary outcome measurements) | yes | no | no | yes | yes | yes | yes | yes | yes | no |
| 7) Explain any patient exclusions from analysis | yes | no | yes | unclear | yes | no | yes | yes | yes | no |
| 8) Describe how confounding was assessed and/or controlled | no | no | yes | yes | no | yes | yes | yes | yes | no |
| 9) If applicable, explain how missing data were handled in the analysis | no | yes | yes | no | no | no | no | no | yes | no |
| 10) Summarize patient response rates and completeness of data collection | no | yes | yes | no | yes | yes | no | yes | yes | no |
| 11) Clarify what follow-up, if any, was expected and the percentage of patients for which incomplete data or follow-up was obtained | no | yes | no | no | yes | yes | no | no | yes | no |
| total points | 7 | 8 | 9 | 7 | 8 | 9 | 8 | 8 | 11 | 5 |
| Remarks:(Yes or no or "unclear" answers;For each question,a "yes" is awarded 1 point;Other answers count as 0 points;The fifth item is the reverse score item;The full score is 11 points,≥8 is classified as high quality,6-7 is classified as medium quality,≤5 is classified as low quality.) | | | | | | | | | | |
|  |  |  |  |  |  |  |  |  |  |  |

As shown in Table 2,among the cross-sectional studies included in this study,7 were of high quality and 2 were of medium quality.1 study had an AHRQ score of 5 and was therefore excluded from this study.
