# Supplementary material for: The relationships between obesity and epilepsy: A systematic review with meta-analysis
Source: PLoS One. 2024 Aug 9;19(8):e0306175. doi: 10.1371/journal.pone.0306175 (PMC11315312; doi:10.1371/journal.pone.0306175)
Supplement: Attachment — Submitted filename: Response to Reviewers.pdf [file pone.0306175.s006.pdf]

Response to Reviewers

Dear editor,

first of all,I would like to thank you and the reviewers for your review of our manuscript and your valuable suggestions for revision.We have carefully read your amendment comments and have carefully revised in response to the issues you have raised.We have marked the revised part of the manuscript in red font,and the specific content can be seen in the "Revised Manuscript with Track Changes".Below are our responses to your comments.

1.Responding to comments from academic editors:

(1)formulation of the aim at the end of introduction section should be done better.emphasize that you are performing meta analysis.It's not enough to say that you are investigating "connection".

Thank you very much for your valuable revision suggestions,so that we can further improve the article.After careful consideration,we have made corrections and additions.We have carefully considered your comments.First of all,we emphasize that we are conducting a meta-analysis,and further explain that the purpose of meta is to better explore the correlation between epilepsy and obesity,and further provide references forclinical diagnosis and treatment of epilepsy.Therefore,our study supplemented the possible influencing factors of obesity and epilepsy,and increased the practical reference value for clinical work.The changes formulated are detailed in the introduction section of the revised manuscript.

(2)emphasize limitation of your study in discussion.

Our study did have some limitations,and we have added a more detailed supplementary note.See the limitations section of the manuscript for details.

(3)make a take a home massage based on your results as a concluding paragraph (giving perspectives are fine,but make a clear conclusion).

I think you're right.We really don't seem to have a clear conclusion.We have added and modified the conclusions in the results section.See the research results sectionof the manuscript for details.

2.Respond to reviewer's comments:

(1)The study was done to examine the correlation between obesity and epilepsy hence only case control studies were selected. I suggest studies of other research design could also be reviewed.

In accordance with your suggestions,we have made careful revisions to our manuscript.Your suggestions are very helpful to improve the quality of our manuscripts.On the basis of the original case-control study,we added cohort studies,non-randomized controlled trials and cross-sectional studies,but after further screening,the cohort studies and non-randomized controlled trials were excluded after they failed to meet our screening conditions.After the final screening,we added a cross-sectional study to further expand our research.This makes our research more comprehensive and convincing.

(2)The result should state the difference that were seen when the sensitivity analysis was done for the studies excluding hightest weight.

We performed a sensitivity analysis by excluding the studies with the highest weight(Fig 5),and the results showed heterogeneity(p=0.008, I^2=52%),indicating that the meta-analysis results may be unstable.

(3)What was the reason behind the subgroup analysis of years of publication and developed and developing countries?

We conducted subgroup analysis of the year of publication and the degree of development of the country in order to find the reasons for the heterogeneity of the meta-analysis results.First,different economic development in different publication years may affect the incidence of obesity and epilepsy.However,subgroups found that publication years were not related to the heterogeneity of meta-analysis results.Secondly,the subgroup analysis of developed and developing countries is also due to the consideration of the impact of economic issues on epilepsy and obesity.However,it is also found that the development level of a country has no significant correlation with the correlation between epilepsy and obesity,and the source of heterogeneity is not found.However,according to your suggestion,we have added some studies and conducted subgroup analysis on the publication years and economic development again,and found that the publication years and economic development level are the sources of heterogeneity.In order to better discuss the source of heterogeneity,we further conducted subgroup analysis and found that age was also the source of heterogeneity in our study.

(4)Also discussion has more part on various drug and their obesity inducing effects which can be tabulated for a better understanding.

Thank you very much for your advice.We have further increased the impact of various anti-epileptic drugs on obesity,which can indeed better understand and compare

the impact of different drugs on obesity,and provide better reference value for clinical drug use.See Table 3 added to the manuscript for details

(5)It would be more comprehensible if the studies are arranged in chronological order as per the year of publication.

Thank you very much for your suggestion,we have revised it.

Finally,thank you again for your valuable comments and those of the reviewers!At the same time,we hope that if you find any deficiencies again in the process of review,please kindly give us your valuable revision suggestions again,and we will continue to revise carefully.We look forward to publishing our articles on your magazine platform,and we will work harder for this.Looking forward to your good news.

Yours sincerely,

Yu-xuan Li

Department of Neurology, The First Affiliated Hospital of Dali University, Dali, China.18408832102

liyuxuandldx@163.com
